# Cost-effective and sustainable operation of microgrids using Improved Whale Optimization Algorithm

**DOI:** 10.1038/s41598-026-35529-y

**Published:** 2026-02-03

**Authors:** Sohayla M. El-Zaher, Aya M. Ahmed, Eman M. Ahmed, Yasmin T. Sedki, Hager K. Al-Muntaser, Ahmed N. Sheta

**Affiliations:** Electrical Engineering Department, Faculty of Engineering, Mansoura University, El- Mansoura, 35516 Egypt

**Keywords:** Microgrids, Energy management system, Improved Whale Optimization, Renewable energy resources, Energy science and technology, Engineering, Mathematics and computing

## Abstract

The global transition to sustainable energy demands efficient integration of renewable resources and resilient operation of microgrids (MGs). This study aims to develop a cost-effective and sustainable Energy Management System (EMS) for MGs operating in both grid-connected and islanded modes. The inherent variability of renewable generation and fluctuating grid prices pose significant challenges to maintaining supply-demand balance. To address this, the proposed EMS employs an Improved Whale Optimization Algorithm (IWOA), incorporating a nonlinear swimming parameter and Lévy flight mechanism to prevent premature convergence. Simulation results on a benchmark low-voltage MG reveal that IWOA achieves a 39.66% reduction in operational costs compared to standard algorithms, while maintaining competitive runtime of 4.2 min. Furthermore, a dynamic energy trading strategy is integrated to optimize real-time interactions with the main grid. The findings validate the proposed framework as a robust solution for enhancing the economic and environmental performance of modern power systems.

## Introduction

Energy plays a central role in global economic development, national security, and industrial and agricultural productivity. As worldwide demand for electrical energy continues to rise, governments face increasing pressure to establish long-term energy strategies that ensure security of supply while addressing environmental concerns. Despite ongoing diversification efforts, the global energy system remains heavily dependent on fossil fuels, resources that are finite and major contributors to carbon dioxide (CO₂) emissions and climate change^1^. The vulnerability of such dependence was made evident during the 1973 global energy crisis, which triggered severe fuel shortages and economic instability. Although the oil embargo ended in 1974, its long-lasting impact demonstrated the fragility of energy systems relying predominantly on non-renewable resources^1^. As of 2019, fossil fuels supplied nearly 80% of global primary energy consumption, and energy-related emissions accounted for approximately 60% of total greenhouse gas emissions^1^. Meeting international climate targets will require reducing CO₂ emissions by more than 70% by 2050, a goal achievable only through accelerated adoption of renewable energy (RE) and improved energy efficiency^2^.

**Fig. 1:**
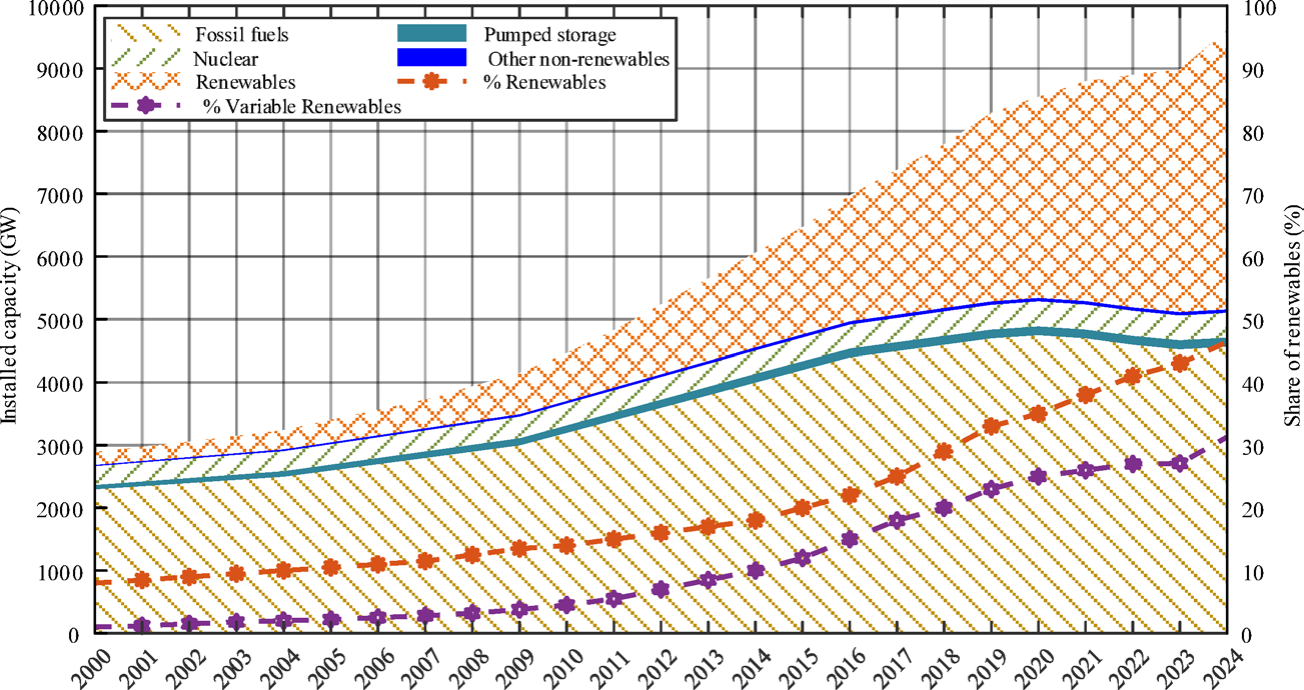
Global installed electricity generation capacity from 2000 to 2024.

Recent statistics highlight the rapid global expansion of renewable energy. By the end of 2024, global installed RE capacity reached approximately 4.448 TW, representing 46.4% of the world’s total power generation capacity, estimated at 9.586 TW^2^. The year 2024 alone witnessed a record-breaking addition of 585 GW of renewable installations, marking the highest annual increase since 2000 and yielding a compound annual growth rate of 10.4% from 2018 to 2023^2^. Variable renewable energy (VRE) sources, primarily wind and solar, experienced the fastest growth, accounting for roughly 31.3% of global power capacity. Solar energy led with 1,865 GW (41.9% of RE capacity), followed by hydropower (1,283 GW; 28.8%) and wind energy (1,133 GW; 25.5%). Bioenergy, geothermal, and marine technologies contributed smaller shares but continue to grow steadily^2^. These trends, depicted in Fig. 1, reinforce the global transition toward cleaner and more sustainable energy systems.

In line with this transition, microgrids (MGs) have emerged as key enablers for integrating distributed energy resources (DERs) and enhancing energy resilience. MGs are localized power systems that incorporate various energy sources and can operate either in grid-connected or islanded modes. Their flexibility makes them well-suited for renewable-rich environments; however, it also introduces operational challenges. These include managing the intermittency of renewable energy sources (RESs), ensuring supply–demand balance, and maintaining system stability under fluctuating load and generation conditions^3^.

To address these challenges, advanced energy management systems (EMSs) have become essential. As defined in IEC 61,970, an EMS supervises the generation, transmission, and distribution of electrical energy to achieve secure, economical, and reliable system operation^4^. Within MGs, EMSs coordinate DER scheduling, manage real-time power flows, support grid interactions, and ensure smooth transitions between operational modes. This requires continuous monitoring, short-term forecasting of renewable generation and load demand, and integration of meteorological and electricity market data^5^. A well-designed EMS enhances the ability of MGs to operate autonomously, improve reliability during grid disturbances, and prioritize renewable resource utilization.

Recent EMS paradigms increasingly adopt multi-objective optimization frameworks aimed at minimizing total operational cost, reducing power losses, and mitigating greenhouse gas emissions. A wide range of techniques has been explored in the literature to achieve these goals, including metaheuristic algorithms such as Genetic Algorithms (GA) and Particle Swarm Optimization (PSO), as well as artificial intelligence (AI) approaches like fuzzy logic, neural networks, and multi-agent systems. In addition, predictive and deterministic methods, such as Model Predictive Control (MPC) and the interior point method, have also shown effectiveness in microgrid (MG) applications^6,7^. More recently, modern AI techniques have gained increasing attention in MG energy management due to their ability to provide adaptive, data-driven decision-making under uncertainty. These methods allow EMS frameworks to optimize scheduling, demand response, and energy trading in real time. In particular, reinforcement learning (RL) has demonstrated strong potential for managing multi-objective dispatch and dynamically coordinating DERs^8–10^. By complementing classical optimization methods, AI-based approaches enhance adaptability to uncertain renewable generation and fluctuating demand, thereby improving the efficiency, resilience, and sustainability of MG operations^11–15^. Overall, these advanced optimization mechanisms, ranging from classical methods to modern AI, are essential for ensuring the operational efficacy, economic viability, and long-term sustainability of MGs. Their application enables MGs to maintain system stability, participate effectively in electricity markets, support ancillary grid services, and strengthen their role in the transition toward resilient, low-carbon energy systems.

Motivated by the need for efficient and environmentally sustainable MG operations, this study proposes the development and optimization of a multi-source MG architecture using an advanced metaheuristic algorithm, the Improved Whale Optimization Algorithm (IWOA). IWOA enhances the original whale optimization algorithm by achieving a more effective balance between global exploration and local exploitation, thereby improving its ability to solve complex, nonlinear, and multi-dimensional problems typical of MG optimization. Its improved convergence characteristics and robustness reduce the likelihood of premature convergence and result in more accurate and reliable solutions under dynamic operating conditions. By integrating IWOA into the EMS framework, the proposed system optimally schedules DERs, minimizes operational costs, and enhances real-time dispatch in both grid-connected and islanded modes while explicitly considering emission reduction objectives. To contextualize the novelty of this work, Table 1 outlines the key features and limitations of the methods examined in the literature, emphasizing the technical gaps that motivate the advanced EMS design and optimization strategy proposed in this study. Generally, the main contributions of this work are summarized as follows:

- Development of an integrated EMS architecture capable of coordinating multiple DERs and ensuring seamless operation across different MG modes, with a strong focus on maximizing renewable utilization.
- Design of an intelligent energy transaction strategy that supports real-time energy exchange with the main grid, enabling optimized purchasing and selling decisions based on market prices, load conditions, and renewable availability.
- Incorporation of emission reduction as a core optimization objective, ensuring that energy-scheduling decisions support both economic performance and environmental sustainability.

The remainder of the paper is organized as follows. Section 2 provides an overview of EMS concepts and functionalities. Section 3 presents the mathematical formulation of the EMS optimization problem for both grid-connected and islanded MG operations. Section 4 discusses simulation results and performance evaluation under multiple scenarios. Section 5 concludes the study and highlights potential future research directions.

**Table 1:** Comparative review of related works and research gaps.

Ref.	Scope / Focus	MG mode	Methodology	Strengths	Limitations / Gaps
^4^	EMS and power forecasting in grid-connected MGs	Grid-connected	Machine learning-based EMS with multiple DERs	Strong forecasting tools; integrates ML for scheduling	Lacks multi-objective optimization; no advanced metaheuristic; no emission objective
^7^	Real-time EMS	Grid-connected	Deterministic optimization for real-time dispatch	Strong for real-time operation	Unable to handle nonlinear multi-source MG complexities
^8^	Automated EMS in rural MGs	Islanded	Deep deterministic policy gradient (DDPG)	Fully automated control; strong adaptability	High computational cost; training instability; lacks multi-objective constraints
^9^	Data-driven MG management	Grid-connected	Review of advanced trends	Highlights modern MG challenges	No solution methodology; no optimization implementation
^10^	Net-zero MG dispatch	Grid-connected	DRL-based business model	Strong long-term policy learning	Not suitable for real-time multi-DER scheduling; no emission minimization
^11^	MG EMS fundamentals	Both	Optimization-based EMS	Good foundational formulation	Outdated; does not reflect modern RES penetration or metaheuristics
^12^	Coordinated regional MG dispatch	Grid-connected	EVs as mobile storage	Incorporates mobile storage	Requires high communication; no advanced metaheuristic optimization

## Energy management system in microgrids

An EMS serves as a centralized, automated, and real-time decision-making platform tailored for optimizing the coordination and scheduling of DERs and controllable loads across islanded and interconnected MG networks. Operating on a large-scale, such as across an entire island or regional distribution network, the EMS integrates various Distributed Generation Systems (DGS) and Energy Storage Systems (ESS), all configured as part of a MG architecture. It performs essential supervisory functions, including real-time data acquisition, system monitoring, and control of all connected MGs to address the complex operational challenges that have arisen in the evolving power sector over the last decade^4^.

The core operational functions of an EMS encompass several interconnected objectives aimed at ensuring efficient, reliable, and environmentally conscious operation of MGs^5^. A primary function is the optimization of energy availability, wherein the EMS maximizes energy access and reliability for end-users while minimizing power losses, reducing operational costs, and improving overall system efficiency. This is achieved through the integration and optimal utilization of renewable energy sources such as solar photovoltaic and wind systems, which also contribute to lowering greenhouse gas emissions and reducing the environmental footprint of energy production. In parallel, the EMS enables economic operation by supporting cost-effective dispatch strategies that decrease dependency on power imports from the main grid, thereby curbing operational expenditures. It also plays a pivotal role in managing the synchronization and resynchronization of DERs, ESS, and controllable loads during transitions between islanded and grid-connected modes, ensuring system stability and seamless operation. To execute these functions effectively, the EMS relies on comprehensive data integration. This includes forecasting information for variable renewable energy generation, load demands, real-time electricity market prices, state of charge (SOC) levels of storage units, and detailed grid operational parameters related to security and reliability. Additionally, real-time data from the point of common coupling (PCC) with the main grid is incorporated to maintain coherent and synchronized system operation^6^.

**Fig. 2:**
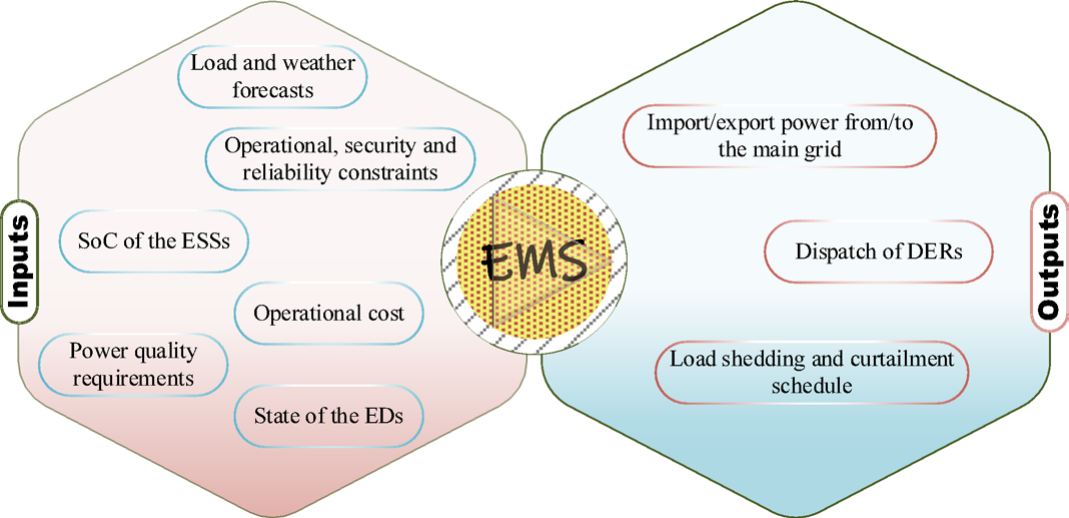
Energy management system diagram.

Using this extensive data infrastructure, the EMS executes advanced control and optimization algorithms. These algorithms are designed to minimize total operational costs, improve the efficiency of energy dispatch across the MG network, and ensure economic energy delivery to end-users. Additionally, the EMS aims to reduce technical losses in the distribution system, improve power quality metrics such as voltage and frequency stability, and enhance the reliability and resiliency of supply under diverse operating conditions^7^. As a result, the EMS generates a hierarchy of control signals tailored for different levels of operation. At the utility level, it issues directives for managing the bidirectional exchange of power with the main grid, regulating both import and export transactions. At the distributed level, the EMS controls individual DERs and ESS units by scheduling their output, managing start-up/shut-down sequences, and overseeing connection or disconnection protocols based on system requirements and contingencies. This layered control framework is illustrated conceptually in Fig. 2^16^.

To ensure the effective implementation of energy management strategies, the integration of a robust and intelligent optimization framework is imperative. Typically, the core of the proposed system is an advanced optimization engine that employs state-of-the-art algorithms to achieve a dynamic and precise balance among energy generation, consumption, and storage. This framework facilitates enhanced coordination and control of DERs, enabling the system to make real-time, data-driven dispatch decisions that prioritize the minimization of operational costs, reduction of greenhouse gas emissions, and enhancement of system reliability and resilience. By systematically addressing the inherent complexities and uncertainties of modern power systems, the framework supports the optimal utilization of available resources while maintaining stability under varying operational conditions. The subsequent section provides a detailed exposition of the adopted optimization methodology, elaborating on the underlying mathematical formulations, algorithmic structures, and control mechanisms employed to achieve substantial improvements in MG efficiency, sustainability, and overall operational performance.

## Multi-objective optimization model for MG-EMS

The rising incorporation of renewable energy sources and the growing complexities of modern power systems have rendered energy management an essential and challenging task^17^. The attainment of a long-term equilibrium between energy supply and demand, the minimization of operating costs, and system reliability maintenance, particularly under conditions of uncertainty and variability become obligatory objectives within this task. Conventional solutions frequently cannot respond completely to these changing requirements, and therefore researchers resort to more advanced and adaptive optimization methods. Recent publications^18^ have compared several methods, including approximate dynamic programming (ADP)^19^, particle swarm optimization (PSO)^20^, genetic algorithm (GA)^21,22^, whale optimization algorithm (WOA)^23,24^, and IWOA^25^. Among the various optimization techniques, the IWOA demonstrates clear advantages, making it particularly suitable for complex multi-objective problems in energy management. Its ability to achieve faster convergence, produce higher-quality solutions, and exhibit enhanced adaptability to multimodal and high-dimensional search spaces allows it to outperform conventional methods in both accuracy and efficiency. Such characteristics are especially valuable in MG systems, where decision-making must balance diverse objectives under uncertain and dynamic operating conditions. In this study, IWOA is applied to optimize the proposed objective function using operational data from two distinct MG operating modes. The algorithm effectively minimizes total operational costs, improves economic returns, and maximizes the utilization of renewable energy resources, thereby supporting both economic and environmental goals. For validation, the performance of IWOA is benchmarked against established optimization methods such as GA, PSO, ADP, and the standard WOA.

### Microgrid mode of operation

**Fig. 3:**
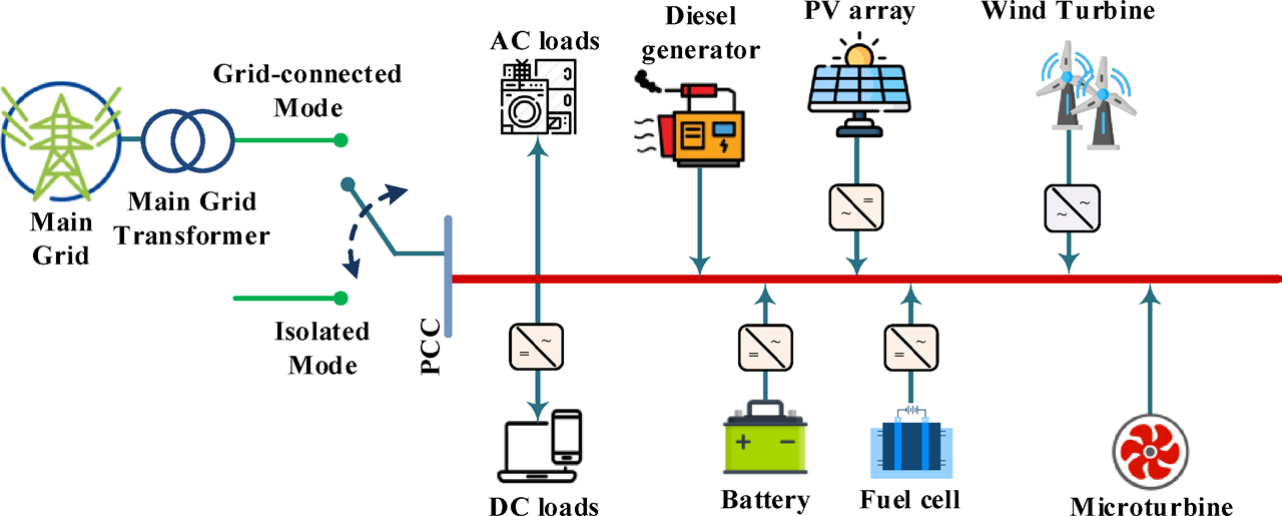
A radial MG layout showing grid-connected and islanded operational modes.

MGs can function in two main modes: grid-connected mode and islanded mode, with different operation characteristics and control needs, as shown in Fig. 3^3^. In grid-connected mode, the MG is synchronized with the main utility grid, enabling bidirectional power flow. This operational mode comprises two primary components: the power supply segment, which encompasses DERs including wind turbines (WTs), photovoltaic systems (PVs), diesel generators (DEs), fuel cells (FCs), microturbines (MTs), and battery energy storage systems (BATs); and the load segment, representing the electrical demand. The MG must maintain dynamic power balance and ensure stable operation of all DERs in this mode. The optimization objectives are to maximize renewable energy source (RES) utilization, minimize interaction with the main electrical network, and reduce both operating costs and pollutant emission costs. Conversely, in islanded mode, the MG operates independently from the main grid, either intentionally (such as during planned maintenance) or due to unforeseen grid outages. The system must rely exclusively on local DERs and energy storage systems to satisfy demand, presenting additional challenges in maintaining frequency and voltage stability. Power generation must continuously match consumption levels without external support, making real-time regulation and effective energy management critical. Both operational modes require sophisticated control strategies and optimization techniques to ensure reliability, economic viability, and environmental sustainability. The system models and objective functions developed in this research address these challenges across both operational paradigms^3^.

#### Optimization problem in islanded mode of operation

In the islanded mode, the objective of optimal operation planning for MGs in islanded mode is to minimize the overall operation costs, including the internal generation costs related to DERs as well as the costs related to pollutant emissions. In this mode of operation, the MG operates independently of the main grid, and there are no transactions related to energy purchase or sale with the utility grid.

##### Objective function

The main objective function can be stated as in (1),

1 $$\:Minimize\:\Rightarrow\:\:F={f}_{1}{\alpha\:}_{1}\:+\:{f}_{2}{\alpha\:}_{2}$$

where $\:F$ is the total cost of the MG in islanded mode, $\:{f}_{1}$ represents the internal operation cost of DERs, and $\:{f}_{2}\:$denotes the pollutant emission cost.$\:\:{\alpha\:}_{1}$and $\:{\alpha\:}_{2}$​ are the weighting coefficients that define the relative priority between economic and environmental objectives. The operational cost function $\:{f}_{1}\:$is defined in (2) and the emission cost function $\:{f}_{2}$ accounts for the environmental penalties due to pollutant emissions from non-renewable DERs and is provided in (3) and (4)^8,9^.

2 $$\:{f}_{1}=\sum\:_{t=1}^{T}\left({C}_{ESS}\left(t\right)+{C}_{DE}\left(t\right)+{C}_{FC}\left(t\right)+{C}_{MT}\left(t\right)\right)$$

where $\:{C}_{ESS}\left(t\right)\:,{C}_{DE}\left(t\right),{C}_{FC}\left(t\right),{C}_{MT}\left(t\right)$ represent the operational costs of the battery energy storage system, diesel engines, fuel cells and microturbines at time $\:t$, respectively.

3 $$\:{f}_{2}=\sum\:_{t=1}^{T}\left({C}_{DE,em}\right(t)+{C}_{FC,em}(t)+{C}_{MT,em}(t\left)\right)$$

where$\:{C}_{DE,em}\left(t\right)\:,\:\:{C}_{FC,em}\left(t\right)\:and\:{C}_{MT,em}\left(t\right)$ are the pollutant emission costs of diesel engines, fuel cells and microturbines at time t, calculated based on the quantities of pollutants emitted (CO_2_, SO_2_, NO_x_, and CO), and the associated unit emission cost for each pollutant, as in Eq. (4):

4 $$\:\left\{\begin{array}{c}{C}_{DE,em}\left(t\right)=\left({E}_{{CO}_{2}}^{DE}+{E}_{CO}^{DE}+{E}_{{SO}_{2}}^{DE}+{E}_{{NO}_{X}}^{DE}\right)\:.\:\:{P}_{DE}\left(t\right)\\\:\:{C}_{FC,em}\left(t\right)=\left({E}_{{CO}_{2}}^{FC}+{E}_{CO}^{FC}+{E}_{{SO}_{2}}^{FC}+{E}_{{NO}_{X}}^{FC}\right)\:.\:\:{P}_{FC}\left(t\right)\\\:{C}_{MT,em}\left(t\right)=\left({E}_{{CO}_{2}}^{MT}+{E}_{CO}^{MT}+{E}_{{SO}_{2}}^{MT}+{E}_{{NO}_{X}}^{MT}\right)\:.\:\:{P}_{MT}\left(t\right)\end{array}\right.$$

where $\:{C}_{DE,em}\left(t\right)\:$is the pollutant emissions cost of a DE at time $\:t$, $\:{C}_{FC,em}(t$) is the pollutant emissions cost of a FC at time $\:t$, and $\:{C}_{MT,em}\left(t\right)$ is the pollutant emissions cost of a MT at time $\:t$.$\:\:{P}_{DE}$, $\:{P}_{FC}$ and $\:{P}_{MT}$ are the output power values of the DE, the FC, and the MT units respectively, at time $\:t$.

##### Cost functions for DERS

- *Diesel engine (DE)*

The DE is an energy production unit that operates by converting diesel fuel into mechanical movement, whose resultant power is then utilized to make electricity-generating generators produce electricity. The technology is unique because it boasts stability in performance, high dependability, and immunity to external interference. In MG configurations, the DE is normally deployed as a backup energy source, complementing to fill power gaps where the output from renewable sources falls short of satisfying the load requirement, the operational cost of a diesel engine is given in^4^.

- *Microturbine (MT)*

The MT is a compact electricity generator powered by a small gas turbine. It is fueled by fuel energy in the form of natural gas or other green fuels. The technology boasts a number of benefits, including high efficiency, low emissions, and operational flexibility. It is particularly well-suited for local energy systems. Microturbines find application in MGs as firm sources of power. They may run continuously or provide backup support when there is a low supply of renewable energy. The operational cost of a micro-turbine is given in^4^.

- *Fuel cell (FC)*

The FC is an electrochemical energy conversion device that generates electricity through a chemical reaction of hydrogen (or other fuel) with oxygen, yet without combustion. It is renowned for high efficiency, low environmental impact, and quiet operation. Fuel cells in MG systems are a clean and reliable source of continuous power, very valuable in reducing emissions and enhancing system sustainability, particularly in conjunction with renewable energy sources. The cost of operating a FC is provided in^4^.

- *Photovoltaic (PV) system*

PV system is an electric power generation technology that directly utilizes light to produce electrical energy by the action of light on semiconductor material through the photovoltaic effect at the junction of semiconductor materials. The power output from the PV system depends on a plethora of factors, including sunlight intensity and PV panel surface temperature and its mathematical expression is provided in^17^.

- *Wind turbine (WT)*

The WT is a power generation technology that converts mechanical energy into electrical energy through wind-powered fan blade rotation. The mathematical expression of wind turbine output power is given in^17^.

- *Energy storage system (ESS)*

The battery storage system also serves as a major element in power supply balance and as an energy buffer. It ensures coordinated control within the MG and stabilizes the interaction between the renewable energy sources and the system. State of charge (SOC) is one of the most important technical parameters to estimate the present energy status of the battery, and its mathematical expression is provided in^16^.

##### Constraints

In the process of optimization model development for an isolated MG, system constraints are central in the assurance of stable, dependable, and secure operation, particularly in conditions when there is no support from the grid. There are technical and operational boundaries in which each of the DERs need to function.

- *Power Balance Constraint*

In islanded mode, the entire power demand should be met internally without depending on the main grid. Therefore, the overall power generated from local resources should be equal to the load demand at any time t. and the constraint is shown in (5):

5 $$\:{P}_{LOAD}={P}_{ESS}+{P}_{PV}+{P}_{WT}+{P}_{MT}+{P}_{FC}+{P}_{DE}$$

where $\:{P}_{LOAD}$ is the total power demand, and each term on the right-hand side represents the power output from ESS, WT, PV, DE, and FC, respectively.

- *Generation capacity Constraint*

Each generation unit must operate within its minimum and maximum output limits. This can be defined as provided in (6):

6 $$\:{P}_{min}\le\:P\_unit\:\le\:{P}_{max}$$

where $\:{P}_{unit}\:$represents the power output of a specific generation unit.

- *Energy Storage Constraint*

The SOC of the energy storage system must remain within safe operating limits, as shown in (7):

7 $$\:{SOC}_{ESS,min}\le\:{SOC}_{ESS}\:\le\:{SOC}_{ESS,max}$$

where SOC represents the current charge level of the storage system.

#### Optimization problem in grid-connected mode of operation

A grid-connected MG is required to preserve a dynamic power balance and the stable operation of the internal power supply system. Although its architecture and operational aspects are mostly identical to those of an islanded MG, significant differences arise in the objective functions and the power balance constraints, thanks to the possibility of power exchange with the main utility grid. Therefore, this study aims to enhance the share of RES, lower the level of dependence on the main grid, and decrease both operational costs and emission-related costs. It is imperative to note that the proposed EMS operates strictly as a tertiary control layer, focusing on optimal economic dispatch and energy scheduling on an hourly basis. As such, the transient dynamics associated with transitions between grid-connected and islanded modes, particularly sub-second voltage and frequency deviations, are assumed to be handled by the primary and secondary control layers. These lower-level controllers are responsible for voltage regulation, frequency synchronization, and maintaining transient stability, ensuring that sub-hourly disturbances are adequately compensated. Within this framework, the EMS provides optimized set-points for power exchange and incorporates generator startup costs to avoid rapid cycling and economic instability. However, detailed transient stability analysis lies beyond the scope of this economic study and is reserved for future work validation. The pertinent system models and related objective functions are explained in the following subsections.

##### Updated objective function

The objective of optimal MG operation planning in grid-connected mode is to minimize the total integrated cost, which includes both the MG’s internal operational costs and the costs associated with pollutant emissions, as illustrated in (8):

8 $$\:Minimize\:\Rightarrow\:\:F={f}_{1}{\alpha\:}_{1}\:+\:{f}_{2}{\alpha\:}_{2}$$

where $\:F\:$is the total cost of the MG in grid-connected, $\:{f}_{1}$ represents the internal operation cost of DERs, and $\:{f}_{2}\:$denotes the pollutant emission cost.$\:\:{\alpha\:}_{1}$and $\:{\alpha\:}_{2}$​ are the weighting coefficients that define the relative priority between economic and environmental objectives. Here, in grid-connected mode, the operation cost function $\:{f}_{1}$ is defined by (9) and (10),

9 $$\:{f}_{1}=\sum\:_{t=1}^{T}\left({C}_{ESS}\left(t\right)+{C}_{DE}\left(t\right)+{C}_{FC}\left(t\right)+{C}_{MT}\left(t\right)+{C}_{grid}\left(t\right)\right)$$

10 $$\:\left\{\begin{array}{c}{C}_{grid}\left(t\right)={C}_{buy}\left(t\right)+{C}_{sell}\left(t\right)\\\:{C}_{buy}\left(t\right)={C}_{b}\left(t\right)\:.{P}_{b}\left(t\right)\\\:{C}_{sell}\left(t\right)={C}_{s}\left(t\right)\:.{P}_{S}\left(t\right)\end{array}\right.$$

where $\:{P}_{b}\left(t\right)\:$and$\:\:{P}_{s}\left(t\right)$ are the amounts of power purchased from or sold to the grid at time t, $\:{C}_{b}\left(t\right)\:$and $\:{C}_{s}\left(t\right)$ are the corresponding unit prices for buying and selling electricity, respectively.

##### Updated power balance constraint

In grid-connected mode, the total power generated locally combined with energy purchased from the grid, must meet the total load demand, as shown in (11):

11 $$\:{P}_{ESS}+{P}_{PV}+{P}_{WT}+{P}_{MT}+{P}_{FC}+{P}_{DE}+{P}_{grid}\:={P}_{LOAD}$$

where $\:{P}_{grid}\:$is power exchanged with the main grid, with a positive value when importing and a negative value when exporting. This constraint guarantees that for each time step, the power accumulated is enough to satisfy system load, considering internal generation and exchange with the grid.

### Improved Whale Optimization Algorithm

**Fig. 4:**
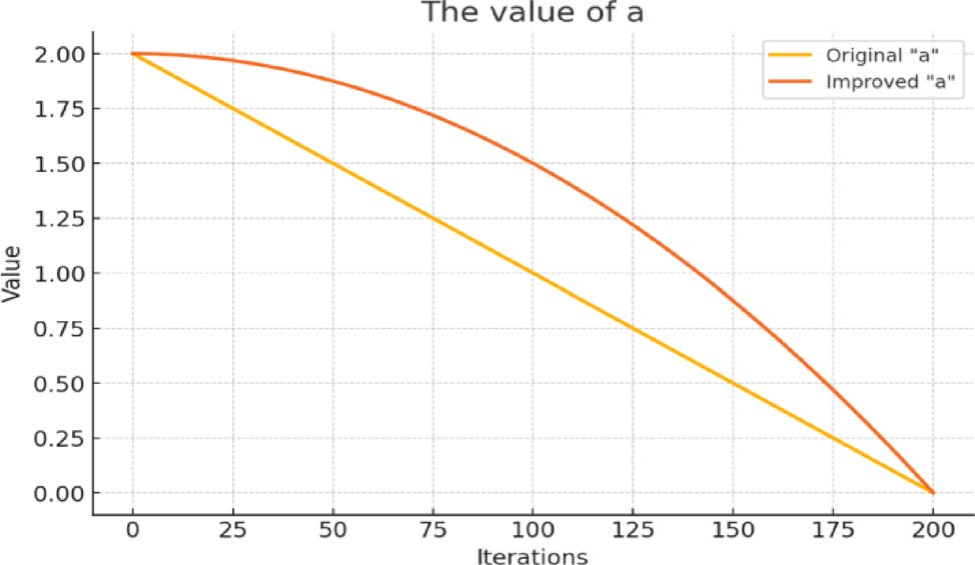
Impact of the swimming factor $\:a$ on optimization model.

MG operation planning is a multi-objective complicated optimization problem with several goals, wherein the primary concern is to determine the power dispatch of DERs while reducing both economic costs and environmental impacts simultaneously. Traditional deterministic and mathematical methods often struggle with this problem due to the nonlinear cost functions of DERs, uncertainty of renewable generation, and diverse operational constraints. In contrast, metaheuristic algorithms such as GA, PSO, and WOA have proven effective in solving non-convex, high-dimensional problems of this nature^18^. In this study, an improved variant of the WOA (IWOA) is adopted to enhance solution quality as well as convergence efficiency. This iteration builds on the standard WOA, first introduced in^23,24^ to mimic the bubble-net hunting method adopted by humpback whales. Although the standard WOA was found to be effective over a variety of optimization problems, it was found to suffer from some limitations such as slow rates of convergence and a tendency to get trapped in local optima, particularly for complicated problem landscapes. To address these issues, IWOA incorporates several enhancements tailored for MG operation planning. First, a nonlinear swimming parameter is employed to dynamically balance global exploration and local exploitation throughout the iterations, mitigating premature convergence in high-dimensional search spaces. Second, an adaptive weighting mechanism refines the exploration–exploitation trade-off over the optimization process, enabling the algorithm to explore widely at early stages while focusing more intensively on promising regions as convergence progresses. Third, opposition-based learning introduces diversity into the search process by considering both candidate solutions and their opposites, thereby expanding the search space and reducing the likelihood of stagnation. Finally, a feedback mechanism leverages the historical performance of candidate solutions to adjust the search trajectory, enhancing stability and reducing oscillations in convergence^25^. Collectively, these improvements dramatically improve the convergence rate, solution quality, and stability of the algorithm, rendering IWOA a strong and reliable solver for multi-objective problems, including MG operation planning^25,26^. The swimming factor ($\:a$) equation in IWOA is given by (12):

12 $$\:a=2{(1-\frac{titer}{{T}_{max}})}^{3}$$

where $\:titer$ represents the current iteration number, $\:{T}_{max}$ is the maximum number of iterations. As seen in Fig. 4, this modification allows the swimming factor $\:a\:$to stay larger at the beginning, improving global search performance, and decrease faster in later iterations, enhancing local search performance.

The implementation of the nonlinear swimming parameter $\:a$ is particularly critical for addressing the temporal coupling constraints. As defined in^4^, the SOC, of the ESS, at any given time step $\:t$ is effectively linked to the previous state $\:t-1$. In standard optimization approaches with linear parameter decay, the algorithm often converges too rapidly, ‘locking in’ a charging/discharging schedule that may be locally optimal for early time steps but suboptimal for the entire 24-hour horizon. By employing the cubic nonlinear decay described in Eq. (12), the IWOA maintains the value of $\:a$ in the higher range for a significantly longer portion of the iterative process compared to standard WOA. This extended exploration phase prevents premature stagnation, allowing the search agents to effectively navigate the multimodal landscape created by the ESS capacity limits and efficiency constraints. Consequently, this ensures that the algorithm discovers a global battery dispatch strategy that maximizes arbitrage opportunities and renewable utilization across the full dispatch period.

Generally, the algorithm employs an adaptive weight during the search phase to make its searching more efficient. The adaptive weight manages the trade-off between global and local search techniques. The algorithm prefers global search when the weight is high, and a lower weight promotes local search. As iterations rise, the weight reduces, enabling the algorithm to explore more cautiously in the domain of the optimum solution. The position updates in IWOA are indicated in (13)–(15):

13 $$\:X\left({t}_{i}+1\right)=X\left({t}_{i}\right)\:+\:A\:\:.\:\:D,\:\:P<0.5\:,\:A<1$$

14 $$\:X\left({t}_{i}+1\right)={X}_{rand}\left({t}_{i}\right)\:+\:A\:\:.\:\:D,\:\:P<0.5\:,\:A\ge\:1$$

15 $$\:X\left({t}_{i}+1\right)=X\left({t}_{i}\right)\:+\:D\:.\:{e}^{b{r}_{2}}\:.\:\mathrm{cos}\left(2\pi\:{r}_{2}\right),\:\:P\ge\:0.5$$

**Fig. 5:**
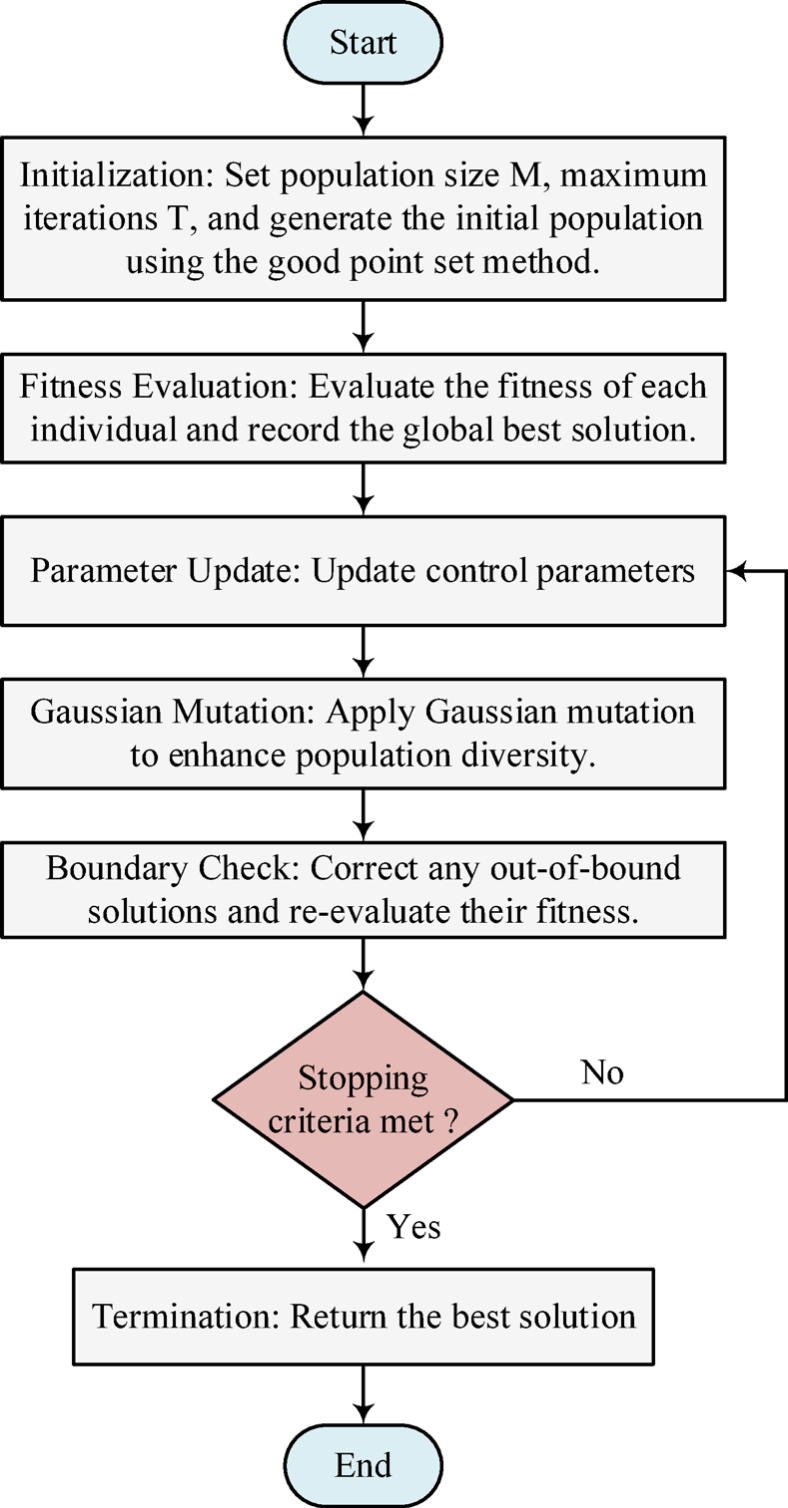
Flowchart of the adopted optimization algorithm.

Where $\:P$ is a random number, $\:A$ is the convergence factor, and $\:D$ is the distance between the current solution and the optimal solution^22,23^. One of the significant changes in IWOA is the integration of Levy flight into the position update process. Levy flight is a random walk with short and long steps, thereby resulting in a larger exploration of the search space. This adaptation enables the algorithm to avoid local optima and increase its possibility of finding global optima. The Levy flight position update is given as (16):

16 $$\:X\left({t}_{i}+1\right)=X\left({t}_{i}\right)\:+\:Levy\:\left({\uplambda\:}\right)\:.\:\:\:X\left({t}_{i}\right)$$

where $\:{\uplambda\:}$ is the step scale factor, $\:Levy\:\left({\uplambda\:}\right)$ represents a random number generated according to the Levy distribution with parameter$\:\:{\uplambda\:}$. Figure 5 outlines the flowchart of the adopted optimization algorithm, while the control parameters employed in this study are summarized in Table 2.

**Table 2:** Control parameters of the IWOA.

Parameter	Value / Range	Role in optimization
Population size	30	Defines number of candidate solutions evaluated in each iteration.
Maximum iterations	500	Termination criterion; ensures sufficient exploration and convergence.
Nonlinear swimming parameter, a	$$\:\mathrm{a}=2{(1-\raisebox{1ex}{$\mathrm{t}\mathrm{i}\mathrm{t}\mathrm{e}\mathrm{r}$}\!\left/\:\!\raisebox{-1ex}{${\mathrm{T}}_{\mathrm{m}\mathrm{a}\mathrm{x}}$}\right.)}^{3}$$	Dynamically balances exploration (global search) and exploitation (local refinement).
Encircling coefficient, A	A = 2a · $\:{r}_{1}$ – a	Controls step size and transition between exploration and exploitation.
Spiral constant, b	b = 1	Determines shape of logarithmic spiral around the best solution for local exploitation.
Adaptive weight factor	*w* (decreases from 0.9 to 0.4)	Speeds up convergence while preserving solution quality.
Lévy flight factor	λ = 0.01	Enables random long jumps to escape local optima and strengthen exploration.

## Results and discussion

**Fig. 6:**
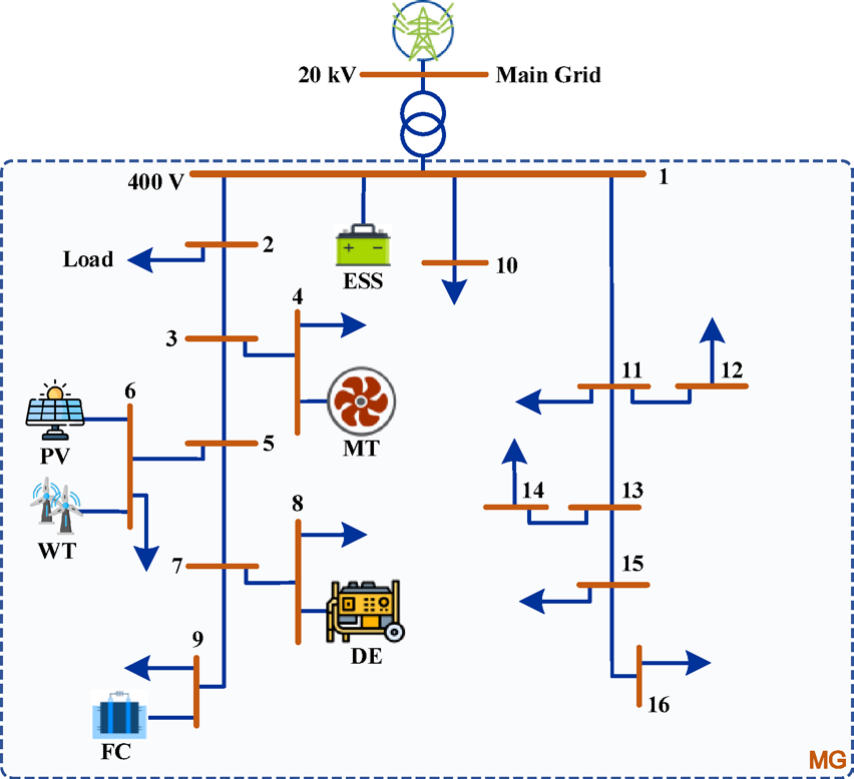
Schematic diagram of the test MG system.

The proposed EMS framework is evaluated using the standard CIGRE Low Voltage Benchmark MG test system, as shown in Fig. 6^27^. The network operates at 400 V and is connected to a 20 kV main grid via a transformer and point of common coupling (PCC). The system integrates various DERs, including a microturbine, fuel cell, wind turbine, photovoltaic panels, battery storage, and multiple loads. The optimal scheduling of these DERs is determined using IWOA, with the technical data detailed in Tables 3, 4, 5 and 6. While this study focuses on a single MG node, the proposed framework is adaptable to larger multi-microgrid configurations. In such expanded topologies, the aggregation of spatially distributed renewable sources typically mitigates intermittency through smoothing effects. Thus, the IWOA is expected to maintain its relative performance advantage in multi-objective formulations involving emissions and reliability indices, provided the population size is adjusted to accommodate the expanded decision variables.

**Table 3:** Generation parameters of each DER in MG.

Type	Minimum power (kW)	Maximum power (kW)
MT	6	30
FC	6	50
DE	1	6
ESS	-30 (negative for charging)	30 (positive for discharging)

**Table 4:** Cost-related parameters of DERs.

Type	b (Ect/kWh)	c (Ect/kWh)	Startup cost
MT	4.37	85.06	9
FC	2.84	255.18	16
DE	2.33	74.3	4

**Table 5:** ESS technical parameters.

Parameters	Value
Charge-discharge efficiency, %	85
Maximum charge-discharge power, kW	30
Maximum state of charge, %	90
Minimum state of charge, %	20
Initial state of charge, %	50

**Table 6:** Gas emission parameters of DERs.

Emission type	Externality cost ($/lb)	FC emission (lb/MWh)	MT emission (lb/MWh)
$$CO_{2}$$	0.014	1.078	1.596
$$SO_{2}$$	0.99	0.006	0.008
$$NO_{x}$$	4.2	0.03	0.44

**Fig. 7:**
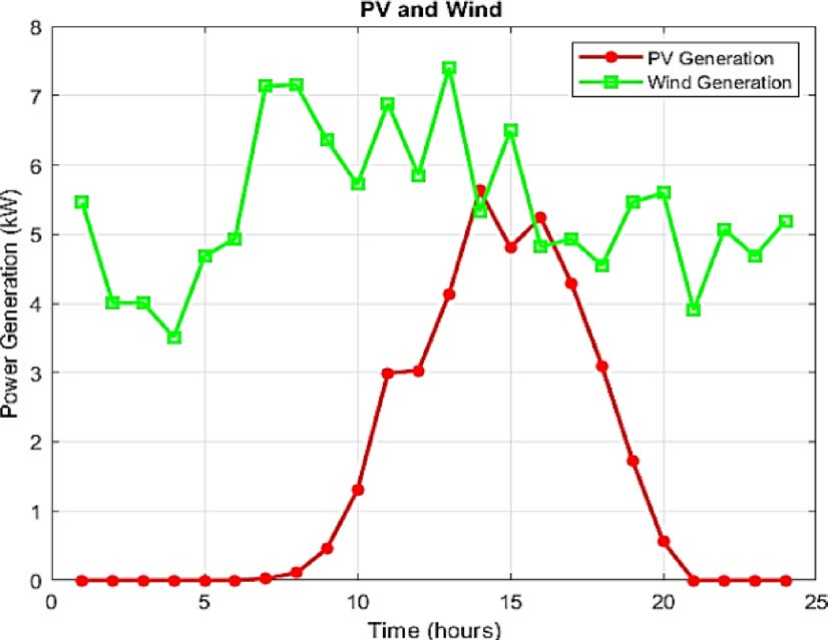
Day-ahead output profiles of renewable/non-dispatchable units.

**Fig. 8:**
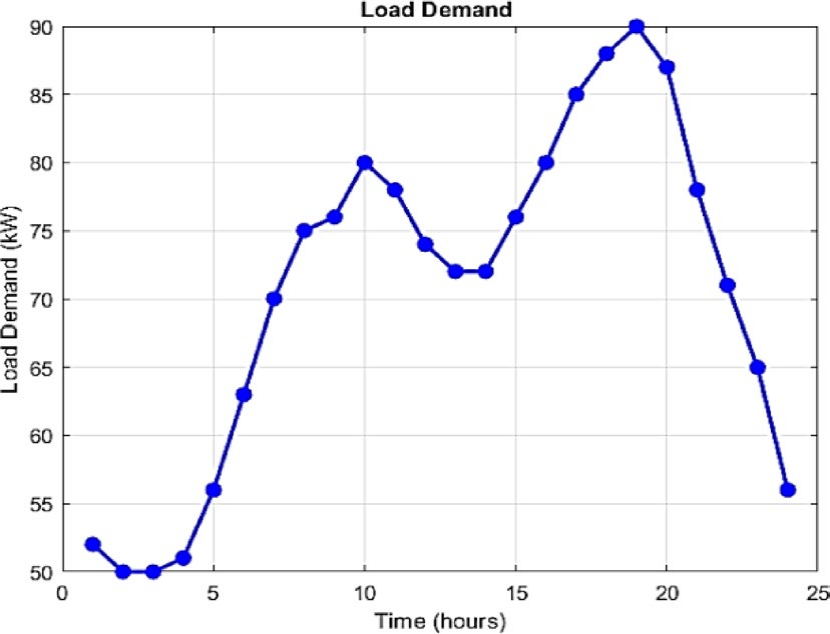
Day-ahead load demand profile.

**Fig. 9:**
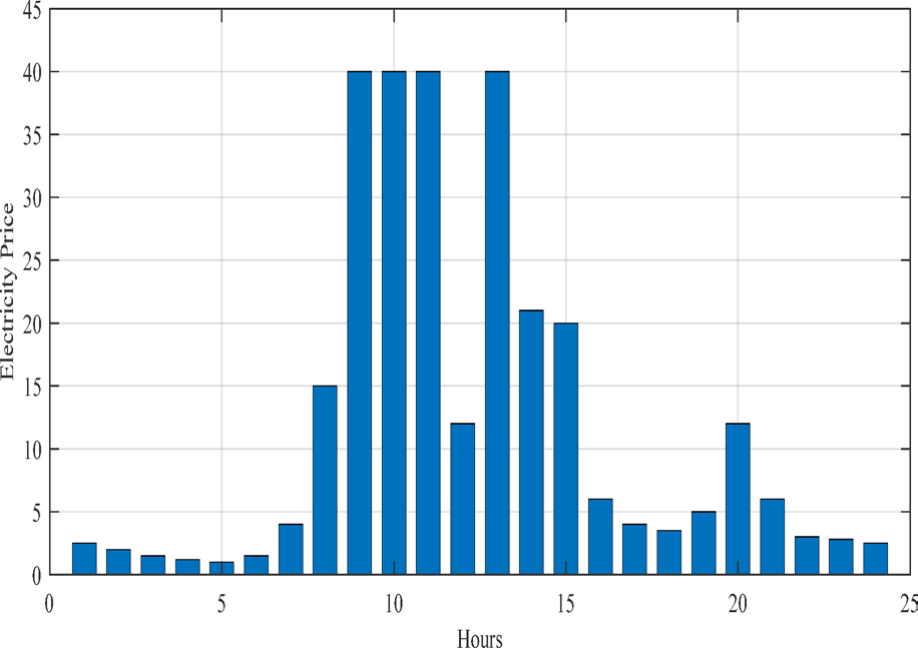
Hourly trading price profile between the MG and main grid.

Notably, in this study, the generation profiles of non-dispatchable units, namely PV and WT systems, are assumed as depicted in Fig. 7. However, future work will incorporate machine learning models to forecast their generation, offering more accurate and adaptive inputs for the EMS. Additionally, the 24-hour electricity demand profile and the main grid exchange price are illustrated in Figs. 8 and 9, respectively. It should be emphasized that these profiles are derived from synthetic test data, which are widely employed in the literature to emulate realistic MG operating conditions^4,16^. The reliance on synthetic datasets is motivated by the limited accessibility of real-world MG data, often restricted due to privacy concerns, proprietary ownership, and availability constraints.

### Islanded mode of operation results

**Fig. 10:**
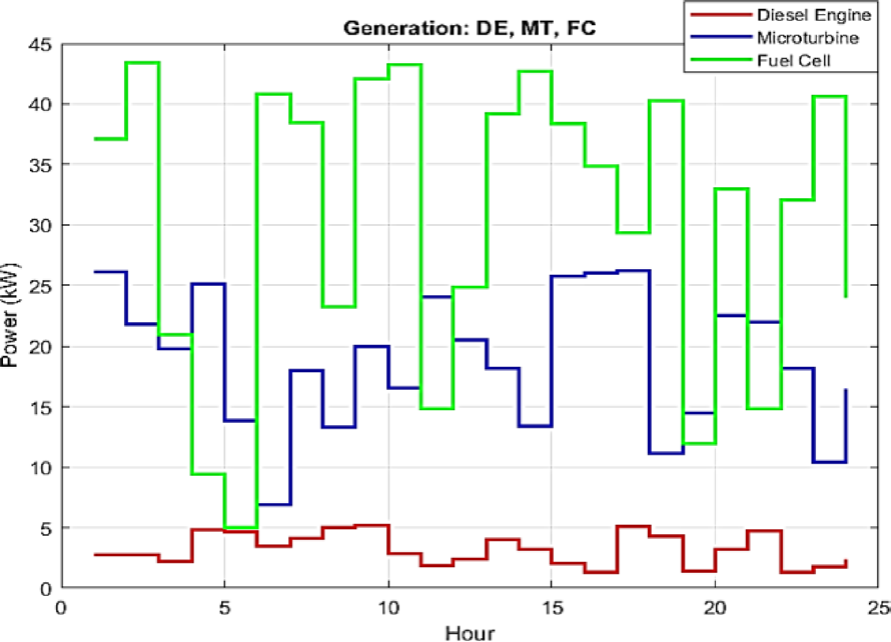
IWOA-optimized scheduling of dispatchable units in islanded mode.

**Fig. 11:**
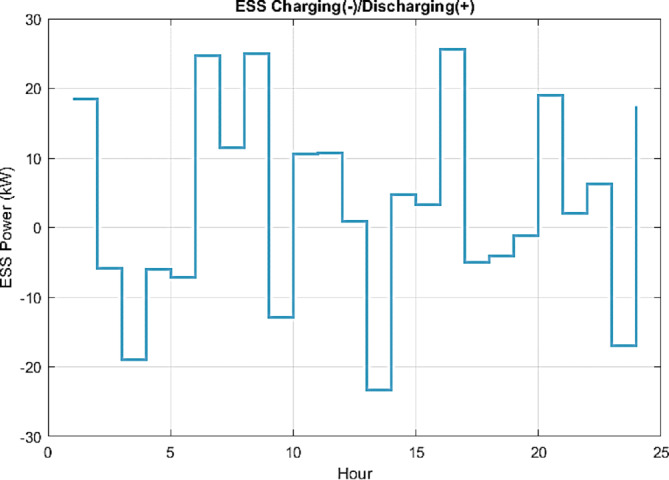
IWOA-optimized scheduling of ESS in islanded mode.

In islanded mode, the maximum load demand is less than the summation of the maximum generation capacity of each unit. Figures 10 and 11 show IWOA-based results obtained for this case.

As depicted in Fig. 10, the dispatch strategy identifies the FC as the primary independent energy resource, attributed to its favorable characteristics such as low operational cost and moderate emission levels. The FC exhibits significant flexibility in its hourly output, ranging from approximately 15 kW to 45 kW, allowing the dispatch algorithm to efficiently adapt to variations in load demand. The MT, in turn, is used as a secondary primary source under constrained dispatch. While the MT offers operational stability and moderate performance, its use involves a trade-off due to relatively higher emissions. Nevertheless, the MT contributes to system flexibility and cost-effectiveness, supporting its role within the hybrid energy mix. In contrast, the DE is engaged only under limited events, operating briefly at low output levels. This minimal reliance indicates the effectiveness of the optimization strategy in reducing dependence on diesel-based generation, mitigating both fuel consumption and environmental impact.

The preference for the FC as the baseload generator is driven by the multi-objective nature of the cost function ($\:F$). Although the DE has competitive fuel coefficients (Table 4), its emission factors for $\:C{O}_{2}$ and $\:N{O}_{x}$ (Table 6) are significantly higher than those of the FC. Since the IWOA optimizes for both operational and emission costs simultaneously, it penalizes the DE, relegating it to a peaking unit utilized only when the FC and MT reach maximum capacity or ramp-rate limits. This confirms that the proposed EMS successfully internalizes environmental externalities into the dispatch logic.

Figure 11 illustrates how the ESS plays a critical role in system balancing by discharging during peak demand periods and charging during low demand or surplus generation intervals. The dispatch strategy incorporates deliberate constraints on ESS power and SOC to ensure compliance with operational limits. The frequent transitions between charging and discharging underscore the ESS’s role as an effective stabilizing mechanism.

Overall, this operational behavior, as discussed above, enhances system stability, particularly in the absence of grid support, while reducing dependence on high-emission generators such as diesel units. Moreover, the dispatch results validate the optimizer’s effective use of the ESS’s flexibility to improve overall system efficiency. The adopted approach in achieving the objective function highlights a deliberate trade-off between operational costs and environmental emissions, resulting in a technically sound, economically viable, and environmentally sustainable operation of the islanded microgrid.

### Grid-connected mode of operation results

**Fig. 12:**
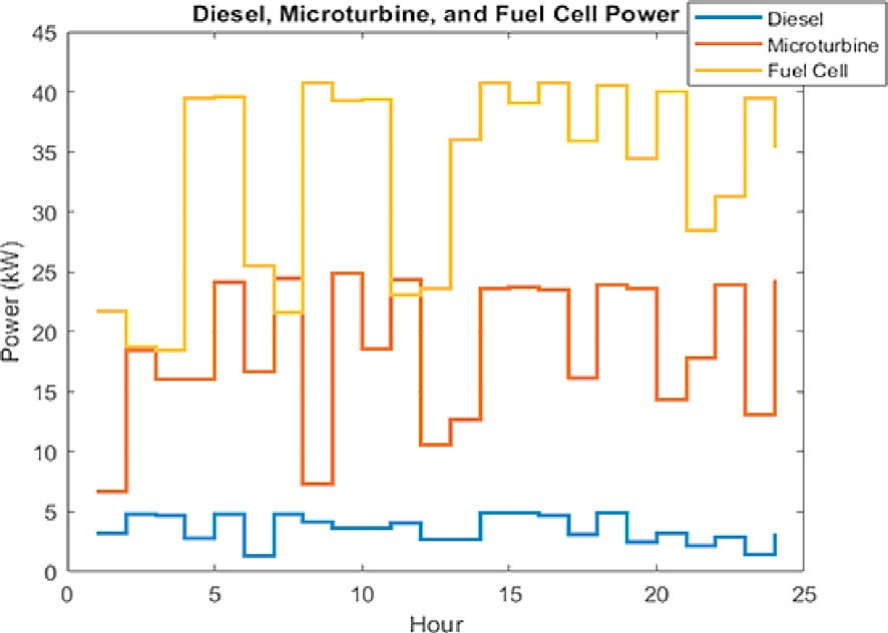
IWOA-optimized scheduling of dispatchable units in grid-connected mode.

**Fig. 13:**
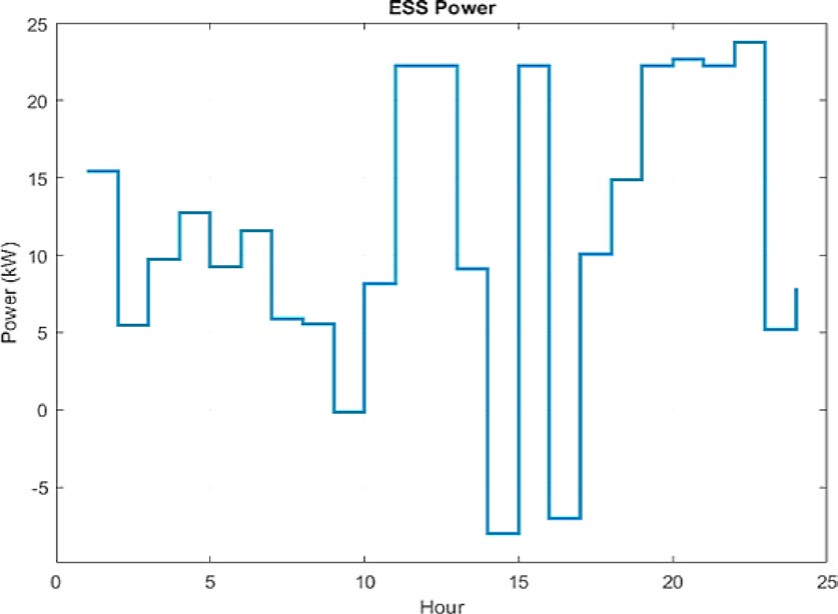
IWOA-optimized scheduling of ESS in grid-connected mode.

In the grid-connected mode, the MG meets its local demand primarily through DERs, while utilizing the main grid to compensate for power shortages or to export surplus energy. Figures 12 and 13 illustrate the optimized power outputs of the DERs and ESS. Figure 12 presents the hourly power output of three conventional DERs: the FC, MT, and DE over a 24-hour period. The FC functions as the primary energy source, operating consistently near its maximum output capacity for most of the day. This reflects its high efficiency and economic advantage as a cleaner alternative. The MT contributes as a secondary source, with its output varying according to demand fluctuations. In contrast, the DE operates intermittently at low output levels, indicating a deliberate effort to minimize its use due to its high emission levels and operational costs. This dispatch strategy demonstrates a prioritized reliance on cleaner and more cost-effective technologies, aligning with objectives of economic and environmental sustainability. Figure 13 illustrates the operational profile of the ESS across the same 24-hour period. The ESS predominantly operates in charging mode, with discharging limited to hours 14, 15, and 17. The deep analysis of the ESS dispatch in Fig. 13 reveals that the IWOA has effectively learned an energy arbitrage strategy. By cross-referencing with the electricity price profile in Fig. 9, it is evident that the discharging intervals (Hours 14, 15, and 17) align with the daily peak pricing windows. Conversely, the ESS charges during the early morning hours (Hours 1–6) when grid electricity prices are at their lowest. This behavior demonstrates that the algorithm is not merely balancing load, but actively minimizing economic costs by shifting demand from high-price to low-price periods, a key capability of smart grid EMS. As such, the system exhibits a dynamic and responsive operation that supports load balancing, enhances energy efficiency, and reduces dependence on high-emission conventional sources.

A critical analysis of these dispatch profiles reveals that the superior performance of IWOA is primarily driven by its refined exploitation mechanism applied to the dispatchable assets, specifically the Fuel Cell and ESS. Since the renewable generation profiles (PV and Wind) are treated as non-dispatchable inputs with fixed maximum power point tracking, the optimization gains are not derived from renewable forecasting adjustments but rather from the precise coordination of the FC and ESS. As evidenced in Fig. 12, the algorithm successfully maintains the FC near its maximum efficiency point (approx. 40–45 kW) to cover baseload, while Fig. 13 demonstrates complex temporal arbitrage where the ESS is aggressively charged during low-price intervals and discharged during peak-price windows. This confirms that the IWOA’s nonlinear convergence strategy effectively navigates the temporal coupling constraints of the storage system, allocating the majority of the optimization effort toward maximizing the economic utility of these controllable resources.

**Fig. 14:**
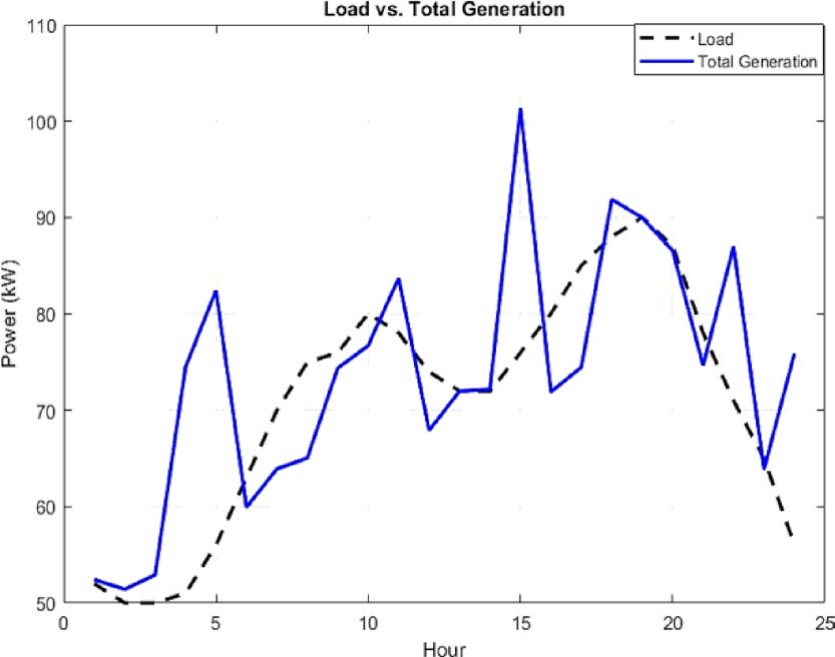
Total generation and load profiles within the MG under grid-connected operation.

**Fig. 15:**
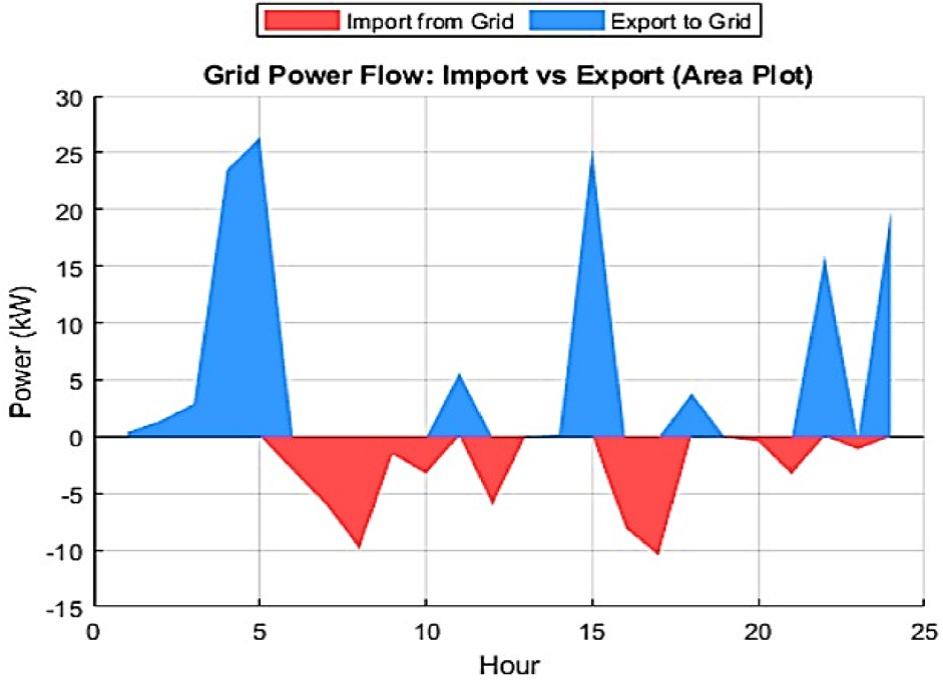
IWOA-optimized MG/main grid net power exchange profile.

Furthermore, to illustrate the power exchange dynamics between the MG and the main grid, Fig. 14 presents the relationship between total generation and system load, identifying the intervals requiring imports from the grid or enabling exports to it. The close alignment of the load and generation curves reflects the system’s ability to minimize energy imbalances and sustain operational stability. The ESS contributes significantly by storing surplus energy during high generation periods and discharging during peak demand as shown in Fig. 13, thereby maximizing self-consumption and reducing operational costs. Finally, Fig. 15 shows the net power exchange between the MG and the main grid, highlighting intervals of both energy import and export. The power exchange dynamics shown in Fig. 15 are directly correlated with the renewable generation profiles. The significant export peak observed between 04:00 and 06:00 corresponds to the high wind generation period shown in Fig. 7, which exceeds the low early-morning load demand. Similarly, the export spike at Hour 15 coincides with the peak solar irradiance. This indicates that the EMS prioritizes self-consumption of renewable energy first, and only exports power when the internal generation from non-dispatchable sources (PV and Wind) creates a surplus that cannot be absorbed by the battery due to SOC constraints or charging power limits. Overall, this bidirectional exchange supports optimal energy utilization, enhances system stability, and strengthens the economic performance of the grid-connected microgrid.

### Comparative evaluation of IWOA against benchmark algorithms

**Fig. 16:**
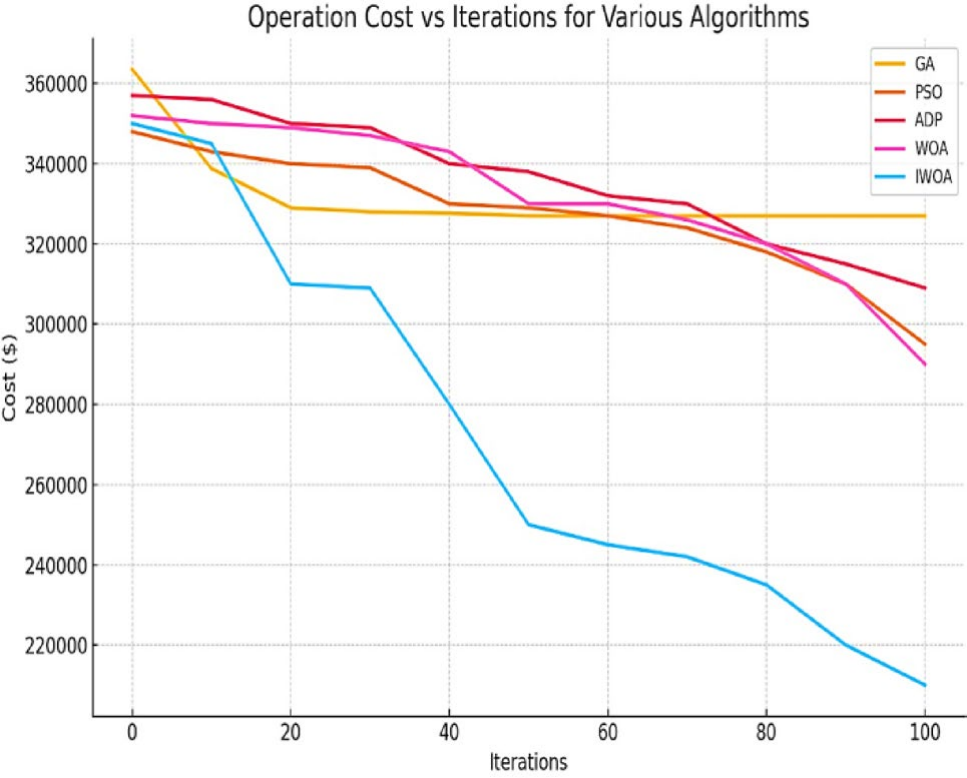
Convergence behavior of various optimization algorithms for minimizing operational cost (objective function).

In this section, the performance of the IWOA is compared against GA, PSO, ADP, and WOA in minimizing the total operational cost of the MG system, as shown in Fig. 16. The convergence characteristics and computational efficiency are summarized in Table 7. The results indicate that GA converges quickly within ~ 20 iterations, but its search process often stagnates at suboptimal solutions, resulting in higher operational costs. PSO demonstrates slightly slower convergence (~ 30 iterations) and achieves better results than GA, yet remains limited by premature convergence. ADP requires more iterations (~ 50) but provides stable solutions at the expense of longer search time. WOA, although computationally inexpensive with the lowest runtime of 3.6 min, converges relatively late (~ 80 iterations) and suffers from reduced solution quality due to its tendency to lose diversity. By contrast, IWOA requires more than 100 iterations to fully converge, with an average runtime of 4.2 min. While this is moderately higher than WOA, the extended search process enables IWOA to maintain population diversity, avoid local minima, and deliver the lowest final operational cost among all tested algorithms. This demonstrates the algorithm’s superior exploration and exploitation balance, making it particularly suitable for addressing the nonlinear, constrained, and dynamic nature of microgrid optimization problems.

These findings align with the established literature^18,21^, which characterizes classical metaheuristics like GA and PSO as prone to premature convergence in high-dimensional multimodal landscapes. Specifically, the stagnation of PSO after 30 iterations (Fig. 16) corroborates the ‘loss of diversity’ phenomenon described in^21^. Conversely, the proposed IWOA extends the search capabilities of the standard WOA^23^ by introducing the nonlinear swimming parameter (Eq. 12). This mechanism successfully forces the algorithm to maintain exploration pressure longer than the standard linear decay allows, effectively addressing the local optima entrapment issues reported in recent microgrid optimization studies^25^.

Furthermore, a critical analysis of the computational complexity reveals that the algorithmic enhancements in IWOA introduce negligible processing overhead. The proposed mechanisms, specifically the nonlinear swimming parameter calculation and adaptive weighting, are scalar operations performed in constant time $\:O$(1). Therefore, the slight increase in average runtime (4.2 min for IWOA versus 3.6 min for WOA) is attributed almost entirely to the extended convergence depth rather than per-iteration computational cost. Unlike GA and PSO, which stagnate early (approx. 20–30 iterations) due to local optima entrapment, IWOA utilizes Lévy flights to sustain population diversity, allowing it to continuously refine the solution beyond 100 iterations. This extended search duration is a deliberate design choice that yields a significant 39.66% cost reduction, validating the trade-off between a marginal increase in runtime and superior solution quality.

**Table 7:** Computational performance of compared optimization algorithms.

Algorithm	Average runtime (minute)	Convergence iterations	Cost reduction percentage (%)
GA	6.8	~ 20	~ 9.67%
PSO	5.9	~ 30	~ 15.71%
ADP	4.5	~ 50	~ 13.45%
WOA	3.6	~ 80	~ 18.08%
IWOA (Proposed)	4.2	> 100	~ 39.66%

To further contextualize the performance of the IWOA, it is constructive to compare it against recent hybrid and advanced metaheuristic frameworks, such as the algorithms presented in^28,29^. While these state-of-the-art methods demonstrate strong optimization capabilities for multi-microgrid coordination and resilience, they often rely on complex hybrid structures that increase computational overhead and parameter tuning complexity. In contrast, the proposed IWOA achieves a competitive 39.66% cost reduction through a streamlined, single-population architecture. This indicates that IWOA offers a superior trade-off between solution quality and implementation simplicity, surpassing the algorithmic efficiency of heavier hybrid counterparts.

Overall, the evaluation confirms that although IWOA involves a longer iterative process, its ability to consistently achieve better-quality solutions with acceptable computational effort highlights its robustness and practical applicability for microgrid energy management.

## Conclusion

This paper presented an integrated EMS framework for robust microgrid operation, optimized using an Improved Whale Optimization Algorithm (IWOA). By introducing a nonlinear swimming parameter and adaptive weighting strategies, the IWOA successfully overcame the premature convergence limitations of traditional metaheuristics. Simulation results on the CIGRE benchmark system confirmed the algorithm’s superiority, achieving a 39.66% reduction in operational costs compared to 18.08% for the standard WOA, alongside significant improvements over GA and PSO.

Beyond the numerical validation, these findings hold significant practical implications for the modernization of distribution networks. Technically, the system’s ability to autonomously export power during renewable peaks and minimize imports during high-price windows demonstrates microgrids can serve as active grid support assets, helping Distribution System Operators (DSOs) reduce congestion and defer costly infrastructure upgrades without centralized control. Economically, the substantial reduction in operational expenditure directly improves the return on investment (ROI) for energy storage systems, addressing a key barrier to microgrid bankability. From a policy perspective, the effective internalization of emission costs within the dispatch logic confirms that regulatory decarbonization targets can be met through algorithmic optimization, prioritizing cleaner generation sources like fuel cells over diesel generators.

Building on the promising results of this study, future work will focus on extending this framework to real-world applications by incorporating machine learning-based forecasting for PV and wind generation. Additionally, the authors plan to conduct a rigorous stochastic robustness analysis and validate transient stability through Hardware-in-the-Loop (HIL) experiments.

## Data Availability

Data will be made available on request from the corresponding author on reasonable request.

## Indices

$t$: Time step index (h)
$titer$: Current iteration number in optimization
${T}_{max}$: Maximum number of iterations

## Decision variables

${P}_{DE}(t)$: Power output of diesel engine at time t (kW)
${P}_{MT}(t)$: Power output of microturbine at time t (kW)
${P}_{FC}(t)$: Power output of fuel cell at time t (kW)
${P}_{ESS}(t)$: Charge or discharge power of ESS (kW)
$SOC(t)$: State of charge of the battery at time t (%)
${P}_{PV}$: Output power of photovoltaic system (kW)
${P}_{WT}$: Output power of wind turbine (kW)
${P}_{grid}(t)$: Power exchanged with the main grid (kW)
${P}_{b}(t)$: Power purchased from the grid (kW)
${P}_{s}(t)$: Power sold to the grid (kW)
${P}_{LOAD}$: Total power demand (kW)

## System parameters

${P}_{min}, {P}_{max}$: Min/max output limits of DER units (kW)
${SOC}_{min}, {SOC}_{max}$: Min/max SOC of ESS (%)

## Cost and economic variables

$$F$$: Total objective function cost ($)
$${f}_{1}$$: Internal operational cost function ($)
$${f}_{2}$$: Pollutant emission cost function ($)
$${C}_{DE}, {C}_{MT}, {C}_{FC}$$: Operational costs of DE, MT, and FC ($)
$${C}_{ESS}$$: Operational cost of energy storage system ($)
$${C}_{DE,em},{ C}_{MT,em}$$: Emission costs for DE and MT ($)
$${C}_{FC,em}$$: Emission cost for FC ($)
$${C}_{buy}$$: Cost of buying electricity from the grid ($)
$${C}_{sell}$$: Revenue from selling electricity to the grid ($)
$${C}_{b}(t), {C}_{s}(t)$$: Unit price for buying/selling electricity ($/kWh)

## Multi-objective parameters

${\alpha }_{1}, {\alpha }_{2}$: Weighting coefficients for objective function

## Algorithmic parameters (IWOA)

$a$: Nonlinear swimming parameter
$$A$$: Convergence factor
$D$: Distance to optimal solution
$\lambda$: Levy flight step scale factor
$P$: Probability control parameter
$b$: Logarithmic spiral constant
*w*: Adaptive weight
$X\left({t}_{i}\right)$: Current position vectors of agents
$X\left({t}_{i}+1\right)$: Updated position vectors of agents
${X}_{rand}\left({t}_{i}\right)$: Random agent position
$Levy \left(\lambda \right)$: Lévy distribution function
${r}_{1}$*,*${r}_{2}$: Random numbers in [0,1]

## Environmental parameters

${E}_{{CO}_{2}}, {E}_{{SO}_{2}}$: Emission factors (lb/MWh)
${E}_{{NO}_{X}}, {E}_{CO}$: Emission factors (lb/MWh)
